# HIV self‐testing: breaking the barriers to uptake of testing among men and adolescents in sub‐Saharan Africa, experiences from STAR demonstration projects in Malawi, Zambia and Zimbabwe

**DOI:** 10.1002/jia2.25244

**Published:** 2019-03-25

**Authors:** Karin Hatzold, Stephano Gudukeya, Miriam N Mutseta, Richard Chilongosi, Mutinta Nalubamba, Chiwawa Nkhoma, Hambweka Munkombwe, Malvern Munjoma, Phillip Mkandawire, Varaidzo Mabhunu, Gina Smith, Ngonidzashe Madidi, Hussein Ahmed, Taurai Kambeu, Petra Stankard, Cheryl C Johnson, Elizabeth L Corbett

**Affiliations:** ^1^ Population Services International (PSI) Washington DC USA; ^2^ Population Services International Harare Zimbabwe; ^3^ Population Services International Blantyre Malawi; ^4^ Society for Family Health Lusaka Zambia; ^5^ Department of HIV World Health Organization Geneva Switzerland; ^6^ Faculty of Infectious and Tropical Diseases London School of Hygiene and Tropical Medicine London United Kingdom; ^7^ Malawi‐Liverpool Wellcome Trust Blantyre Malawi

**Keywords:** HIV self‐testing, HIV testing, men, adolescents, stigma, Malawi, Zambia, Zimbabwe

## Abstract

**Introduction:**

Social, structural and systems barriers inhibit uptake of HIV testing. HIV self‐testing (HIVST) has shown promising uptake by otherwise underserved priority groups including men, young people and first‐time testers. Here, we use characteristics of HIVST kit recipients to investigate delivery to these priority groups during HIVST scale‐up in three African countries.

**Methods:**

Kit distributors collected individual‐level age, sex and testing history from all clients. These data were aggregated and analysed by country (Malawi, Zambia and Zimbabwe) for five distribution models: local community‐based distributor (CBD: door‐to‐door, street and local venues), workplace distribution (WD), integration into HIV testing services (IHTS), or public health facilities (IPHF) and during demand creation for voluntary male medical circumcision (VMMC). Used kits were collected and re‐read from CBD and IHTS recipients.

**Results:**

Between May 2015 and July 2017, 628,705 HIVST kits were distributed in Malawi (172,830), Zambia (190,787) and Zimbabwe (265,091). Community‐based models, the first to be established, accounted for 519,658 (82.7%) of kits distributed, with 275,419 (53.0%) used kits returned. Subsequent model diversification delivered 54,453 (8.7%) test‐kits through IHTS, 23,561 (3.7%) through VMMC, 21,183 (3.4%) through IPHF and 9850 (1.7%) through WD. Men took 294,508 (48.2%) kits, and 263,073 (43.1%) went to young people (16 to 24 years). A higher proportion of male self‐testers (65,577; 22.3%) were first‐time testers than women (54,096; 17.1%) with this apparent in Zimbabwe (16.2% vs. 11.4%), Zambia (25.4% vs. 17.7%) and Malawi (27.9% vs. 25.9%). The highest proportions of first‐time testers were in young (16 to 24 years) and older (>50 years) men (country‐ranges: 18.7% to 35.9% and 13.8% to 26.8% respectively). Most IHTS clients opted for HIVST in preference to standard HTS in each of 12 delivery sites, with those selecting HIVST having lower HIV prevalence, potentially due to self‐selection.

**Conclusions:**

HIVST delivered at scale using several different models reached a high proportion of men, young people and first‐time testers in Malawi, Zambia and Zimbabwe, some of whom may not have tested otherwise. As men and young people have limited uptake under standard facility‐and community‐based HIV testing, innovative male‐ and youth‐sensitive approaches like HIVST may be essential to reaching UNAIDS fast‐track targets for 2020.

## 1 Introduction

In 2016, 36.7 million people were living with HIV (PLHIV), with 1.8 million new HIV infections and one million HIV/AIDS‐related deaths 1. Despite substantial progress toward the 2020 “90/90/90 targets” current estimates suggest we are already off‐track 2, with only an estimated 75% [55% to 92%] of PLHIV currently aware of their status 3. This gap compromises the whole cascade, and also threatens global HIV prevention targets. By 2020, three million high‐risk people should be accessing pre‐exposure prophylaxis and 25 million men provided with voluntary medical male circumcision (VMMC) in 14 African countries 4.

Low HIV testing, knowledge of status, and suboptimal treatment and prevention coverage among men and young people (15 to 24 years) in sub‐Saharan Africa are key gaps in the HIV response. Recent population‐based HIV impact assessments (PHIA) in Zimbabwe, Malawi and Zambia, showed that men with HIV were less likely to know their status than HIV‐positive women 5, 6, 7. Less than half of youth aged 15 to 24 years with HIV knew their status, which was substantially lower than coverage in older age groups 5, 6, 7. Demographic and health surveys (DHS), conducted in 30 sub‐Saharan African countries during 2011 to 2016, showed lower testing coverage among men compared to women for all age groups except 45‐ to 49‐year olds 3.

Low coverage (defined as the proportion of population eligible for an intervention that has received it) of HIV testing and treatment among men in Africa is often due to poor utilization of public sector health facilities, reflecting both social and structural health systems barriers 8, 9. Prevailing social norms around masculinity that emphasize toughness, self‐reliance and sexual success lead to an avoidance of health services, among other consequences 10, 11, 12, 13. For HIV, this is compounded by anticipated loss of social standing and sexual desirability if diagnosed HIV‐positive, increasing fear of stigma and promoting a mindset whereby testing when “still healthy” is considered undesirable 10, 13. Greater formal and informal employment among men compared to women, can also hinder access due to job insecurity and high opportunity and indirect costs 13. Likewise, young people have well‐described age‐specific barriers that make use of existing facility‐based HIV testing services especially difficult 14. Recognizing and responding with innovative male‐ and youth‐sensitive approaches is likely to be an essential component to reaching UNAIDS fast‐track targets for 2020.

HIV self‐testing (HIVST) appeals to the very people left behind by existing HIV testing services (HTS), including young people (15 to 24 years), adult men, key populations (men who have sex with men, people who inject drugs, people in prisons and other closed settings, sex workers and transgender people) and partners of people living with HIV (PLHIV). HIVST provides an empowering opportunity for individuals to test when, where and with whom they want to 14. The ability to test in private and having more control over the testing process have been cited as key motivators to self‐test particularly among men and young people 13, 14. When followed by timely uptake of prevention and treatment services, HIVST can be a key element in the push towards ending AIDS 15, 16. While previous studies have reported on preferences and uptake of HIVST, there has yet to be a multi‐country investigation into the impact of alternative distribution and linkage strategies to optimize testing, VMMC and treatment after HIVST among men. Here, we present quantitative programme data from different HIVST distribution models.

## 2 Methods

Distribution models are summarized in Table 1, with five main approaches described below. OraQuick HIV Self‐Test (OraSure Technologies LLC, Bethlehem, PA, USA) kits were distributed in all countries. Data reported here relate to the first 15 months of distribution (May 2016 to July 2017).

**Table 1:** Summary of models of distribution

Model	Target population	Distribution model description	Rationale
1. Community based (mainly door‐to‐door)	Rural populations: esp. adult men, young people (16 to 24 years) unable to access conventional testing services	HIVST kits offered at household by CBD for clients to test on own or with assistance. Referral facilitation by CBD for confirmatory testing, ART, and prevention services	Increases testing in populations who would otherwise not seek testing services, rapidly and drastically increases testing coverage
2. HIVST integrated into Mobile Services or HIVST fixed sites	High risk adults, adult men (>25 years), adolescents 16 to 24, esp. girls & young women	Distribution at community hotspots e.g. shopping centres, taxi ranks, urban and rural hot‐spots (bus or truck stops, growth points). Confirmatory testing and in some cases ART on site	Test‐for‐ triage: fast track pre‐screening, triaging out those who self‐test HIV negative unless confirmatory testing desired. Providers shift in attention: to those who require more attention and increasing: – index testing and assisted partner notification, confirmative testing of HIV positives, initiation of ART
		People can test themselves in a cubicle at the distribution point or HTS Clinic (with assistance available) or take kit home	Increase in demand for HTS, if mobile services or fixed HTS clinic services are promoted as outlets for HIVST kits
	Sexual partners of HIV+ index diagnosed at HTS (secondary distribution)	HIVST kit offered to HIV+ index to take to sexual partner(s). Follow up with index or partner for confirmative testing	Increases likelihood of sexual partner to take up HIV testing. Based on evidence high proportion of sexual partners of positive indexes are testing positive
3. HIVST offered at male dominated workplaces	High risk adults, adult men (>20 years)	HIVST kits are offered to employees at male dominated workplaces after buy‐in and agreement has been obtained from the employer. Employees can choose to perform HIVST in a private space provided at the workplace where assistance is available or take the HIVST kit home	Increases testing in populations who would otherwise not seek testing services, rapidly and drastically increases testing coverage
4. Integrated with public sector facility	Patients accessing health care facilities in urban and rural areas	Facility‐based counsellors and Health care workers are directly promoting HIVST at entry points of the health delivery system, e.g. outpatients, in‐patients	Test‐for‐triage approach and HTS clinic shift in attention (as above) Increases numbers tested, and coverage of more targeted provider‐initiated testing to maximize HIV diagnoses, ART initiation and prevention service uptake
	Sexual partners of HIV+ index diagnosed at HTS (secondary distribution)	HIVST kit offered to HIV+ index to take to sexual partner(s). Follow‐up with index or partner for confirmative testing	Increases likelihood of sexual partner to take up HIV testing. Based on evidence high proportion of sexual partners of positive indexes are testing positive
	Male partners of pregnant women accessing public sector maternity services (secondary distribution)	HIVST kit is offered to all pregnant women regardless of HIV status to take to male partner	Increases the opportunity of male sexual partners of pregnant women to access HIV testing services, and to be linked to care, treatment and prevention, dependant of status
5. Integration with VMMC Mobilization	Adult males, 20 and above, who are mobilized for VMMC services	HIVST is offered to adult males, who are mobilized for VMMC, to use at home before accessing VMMC services	Fear of a positive test result and fear of testing prevents adult males from taking up VMMC services
			Offering HIVST can reduce this barrier and increase motivation to take up VMMC

Social harms monitoring systems were part of all distribution models. No suicides were identified and reports of other serious harms (potential life‐threatening/life‐changing) were rare (1 event per 10,000 HIVST kits distributed), as discussed in detail for Malawi in this JAIS Special Issue 17.

### 2.1 Model 1: community‐based HIVST distribution

Community‐based distributors (CBDs) provided HIVST kits across 53 districts in Malawi, Zambia and Zimbabwe. Models are described in detail elsewhere 18, 19, 20. In brief, CBDs needed to have completed secondary school education and be resident in the distribution community. CDB recruitment used participatory approaches with candidates nominated following community sensitization meetings. CBDs completed a two‐day training provided by Population Services International (PSI) including basic facts about HIV transmission and treatment, antibody‐based diagnosis, discordancy and the principles of consent and confidentiality, as well as familiarization with the kits and how to demonstrate use to recipients, and data capture tools. All trainees had to undergo competency testing at the end of the training course when training skills were assessed. CBDs promoted and offered free HIVST kits for use alone or with CBD support. The same methods were used by CBDs to offer HIVST kits in households and social venues such as market places, busy streets, bars and beer halls. Individuals could also collect kits from the CBDs home at any time, if preferred.

CBDs provided all clients with brief health information about HIV, information on the test, and an in‐person or video‐clip demonstration‐of‐use and instructional materials optimized for local use demonstration to supplement manufacturer's instructions‐for‐use that were available in local languages.

Clients could choose to self‐test alone, or with the CBD, and were asked to return their used kit and results in a sealed envelope, together with a short, self‐administered questionnaire (SAQ) in collection boxes at community locations. Illiterate and semi‐literate participants were supported by the CBD who was reading out the questions and answers from the SAQ with participants then left to complete the check‐box answers in private.

Additional post‐test guidance was available from CBDs on demand. All self‐testers received self‐referral cards with several locally adapted options to facilitate results‐based linkage into HIV care and prevention services. CBDs collected information on social harms related to HIVST and referred clients for additional management as needed. A toll‐free hotline was available to answer questions about the testing process, results and referral options.

### 2.2 Model 2: HIVST integration into PSI‐led HTS facilities and mobile HTS outreach

Integrated HIVST was piloted from June 2016 and scaled‐up from January 2017 as an alternative option to provider‐delivered testing for clients attending existing PSI‐led HTS clinics and 11 mobile outreach sites in Zimbabwe. Outreach sites included “hot spots” at bus and truck stops, mining areas and urban shopping malls, and other informal workplaces. The aim was to expand choice and support efficiency gains by integrating HIVST with conventional HTS. An additional four months of detailed distribution site data are included here (through November 2017).

After registration, HTS clients were offered a kit that they could use for HIVST on‐site or at home. Clients opting for HIVST received a brief demonstration either by video or by a trained provider. Clients opting out of HIVST received conventional HTS. Private cubicles or tents, with offer of counsellor assistance, were provided to those self‐testing on site. On‐site confirmative testing was available for those reporting a reactive (positive) self‐test result. If confirmed, PLHIV were referred for ART according to national guidelines, with immediate initiation if ART services were either available onsite, or through a referral form to ART services at public and private sector health care facilities.

All clients opting for HIVST received information about post‐test support services and referral forms (confirmative testing and HIV treatment including ART for those with reactive results, information about prevention services for those with negative HIVST results) prior to HIVST. Men were encouraged to consider VMMC if they tested negative, and condom use was promoted. Clients who decided to self‐test at home received information materials listing local prevention and treatment services, and a self‐referral form suitable for either prevention or ART services, dependant on HIVST result.

HIV positive index clients diagnosed at the HTS site were offered self‐test kits for secondary distribution to all their sexual partners for the purposes of index‐testing 21. Clients taking kits for secondary distribution were talked through the process of supporting their partner to use and interpret the kit correctly, how to access follow‐on HIV services, and the need to maintain voluntariness 22.

Self‐testers were asked to leave their used test kits with an SAQ in sealed envelopes at the site, while provider‐delivered HTS clients had data captured by the counsellor. Used self‐test kits were re‐read by the providers on the same day, with this approach used to estimate the number and proportion of HIV‐positive self‐tests.

### 2.3 Model 3: HIVST distribution at workplaces

At larger male dominated workplaces in the mining and farming industry, HIVST kits were distributed through peer‐promotors or PSI HTS outreach workers, who provided pre‐test information and in‐person demonstrations of the self‐testing process. Clients could self‐test on site or at home and could take a test kit home for their partner to use, with support for secondary distribution as described above. Confirmatory testing was available on site, provided by the PSI HTS outreach team or by workplace HTS services, or through self‐referral forms providing information on local private and public‐sector health services. Confirmed PLHIV were referred for ART at public or private sector providers. A toll‐free hotline number was provided to all clients.

### 2.4 Model 4: HIVST distribution at public sector health facilities

Patients accessing public sector outpatient departments (OPD) or other clinical services were offered HIVST by healthcare providers, either nurses or counsellors working at OPD, before their consultation. Clients could self‐test in a separate room following a brief demonstration, with the option of sharing their results during their consultation. Information on confirmatory testing, ART and HIV prevention services was provided to all patients. For those with positive self‐tests, counselling, confirmatory testing and ART were available on‐site through the routine facility services. HIVST‐negative clients received HIV prevention messages by the nurse and healthcare provider in OPD and male clients were referred for VMMC.

### 2.5 Model 5: HIVST integrated with VMMC promotion

VMMC was already being rolled‐out in all three countries by PSI, and HIVST was integrated into mobilization strategies. VMMC mobilizers were trained to offer HIVST to all men who were interested in circumcision, but cited fear of HIV testing onsite. VMMC mobilizers, who had all received a two‐day training course, as described for the CBDs, provided pre‐test information and demonstration of kit use before offering a kit to each potential VMMC client. In Zambia, VMMC mobilizers also distributed HIVST kits to women.

### 2.6 Data collection and analysis

HIVST kit distributors collected individual‐level demographic and HIV testing history data from all clients, using either electronic or paper‐based forms. Data from SAQs were entered into databases at country‐level. Data were aggregated and presented by distribution model at PSI central level. STAR HIVST programme data from Malawi, Zambia and Zimbabwe was analysed according to age, sex, distribution model, testing history and compared between countries. We also compared characteristics of clients, including HIV result and number of HIV‐positives identified, who took up the offer of HIVST with those of clients preferring provider‐delivered HTS at PSI‐led facilities and mobile outreach services. Given the high numbers of testing events (making standard p‐values uninformative), and the intrinsic clustering nature of data from different sites, we present data descriptively without use of testing for statistical significance.

### 2.7 Ethical considerations

All HIVST kits distributed before July 2017 were covered by country‐level research protocols approved by the Ethics Committees of London School of Hygiene and Tropical Medicine, and the relevant ethics committees in Malawi, Zambia and Zimbabwe. As a public health intervention using a version of an HIVST product already approved for over‐the‐counter sale in USA and shown to have minimal potential for harm in Malawi, approved protocols included request for waiver of written or verbal informed consent for HIVST clients. Clients were instead informed about the investigational nature of the HIVST kit through community sensitization events, information leaflets and marking of kits as for research purposes only.

## 3 Results

A total of 628,705 HIVST kits were distributed in Malawi (172,830), Zambia (190,787) and Zimbabwe (265,091). The breakdown of distribution in each country under different models is shown in Table 2, together with the gender, age‐group and numbers of first‐time testers.

### 3.1 HIVST distribution models and client characteristics

Community‐based distribution by CBDs had already been established in Malawi as a model that was acceptable and could support accurate HIVST use and linkage to HIV care services with minimal social harms 23 and was the first model taken to scale in each country. The CBD model accounted for 94.5% of test kits distributed in Malawi, 82.2% in Zambia and 75.3% in Zimbabwe.

Other models were delayed by need for initial piloting, and some (notably HTS integration and VMMC demand creation) were also dependent on the scope and scale of suitable PSI programmes, which varied country‐to‐country. In this respect, Zimbabwe‐PSI had a large HIV service provision platform from which to rapidly diversify and scale‐up HIVST models based on integration into fixed and outreach teams already providing HTS, accounting for 52,254 of the 54,453 kits distributed using this model to July 2017. Similarly, in Zambia, the large pre‐existing VMMC programmes supported rapid scale‐up of HIV delivered through VMMC mobilizers (15,092), with Zambia also leading on integration of self‐testing into public sector clinics.

Nearly half of HIVST kit recipients (294,502; 48.2%) were men (49.0% in Malawi, 50.7% in Zambia and 46.2% in Zimbabwe), and 263,973 (43.1%) were in the 16 to 24‐year‐old age‐group (50.8% in Malawi, 48.9% in Zambia and 34.3% in Zimbabwe).

### 3.2 Reach to first‐time testers

The overall proportion of first‐time testers (Tables 2 and 3) was 19.6% (119,673), varying from 26.8% in Malawi, to 21.6% in Zambia, to 13.6% in Zimbabwe (where self‐testing was introduced to communities previously served by standard HTS delivered by mobile outreach teams). A higher proportion of men (overall 22.3%) than women (overall 17.1%) were first‐time testers in each of the three countries.

**Table 2:** Number and percentage of HIVST kits distributed by country, distribution model, age, sex and previous testing history (first‐time testing)

	Malawi		Zambia		Zimbabwe		Total	
All distribution models	172,830	100.0%	190,784	100.0%	265,091	100.0%	628,705	100.0%
Community‐based distributors	163,300	94.5%	156,806	82.2%	199,552	75.3%	519,658	82.7%
HTS integration (seven months)	2,199	1.3%			52,254	19.7%	54,453	8.7%
Work place	6,004	3.5%	298	0.2%	3,548	1.3%	9,850	1.6%
Public sector			18,588	9.7%	2,595	1.0%	21,183	3.4%
VMMC demand creation	1,327	0.8%	15,092	7.9%	7,142	2.7%	23,561	3.7%
Demographics available^a^	172,830		172,562		265,091		610,483	
Male sex	84,603	49.0%	87,418	50.7%	122,487	46.2%	294,508	48.2%
Age group								
16 to 24	87,744	50.8%	84,437	48.9%	90,892	34.3%	263,073	43.1%
25 to 34	45,864	26.5%	50,168	29.1%	61,438	23.2%	157,470	25.8%
35 to 49	29,405	17.0%	28,926	16.8%	55,464	20.9%	113,795	18.6%
50+	9,817	5.7%	9,031	5.2%	57,298	21.6%	76,146	12.5%
First time testers	46,402	26.8%	37,232	21.6%	36,039	13.6%	119,673	19.6%
Men	23,585	27.9%	22,180	25.4%	19,812	16.2%	65,577	22.3%
Women	22,817	25.9%	15,052	17.7%	16,227	11.4%	54,096	17.1%

HIVST, HIV self‐testing.

a Demographic data were available for all HIVST test users in Zimbabwe and Malawi. For Zambia, demographic data were available for 172,562/190,784 self‐test users.

**Table 3:** Number and percentage of HIVST kits distributed by sex and age for all tested and for all first‐time testers

	All self‐tested	First‐time testers
	N	N (%)
Men	294,508	65,577 (22.3%)
Age group		
16 to 24	130,223	38,295 (29.4%)
25 to 34	78,268	12,800 (16.4%)
35 to 49	55,345	9,226 (16.7%)
50+	30,672	5,256 (17.1%)
Women	315,976	54,096 (17.1%)
Age group		
16 to 24	132,850	32,456 (24.4%)
25 to 34	79,202	8,370 (10.6%)
35 to 49	58,450	6,384 (10.9%)
50‐plus	45,474	6,886 (15.1%)

HIVST, HIV self‐testing.

A further breakdown of the proportion of all self‐testers who were first‐time testers is shown for men and women by age‐group in Table 3. This shows higher proportions of first‐time testers in the youngest age‐group for both young men (29.4%) and women (24.4%), but with a substantial minority of clients in the older age‐groups for both men (16.4% to 17.1%) and women (10.6% to 15.1%).

### 3.3 Community‐based distribution model

The CBD model was evaluated in detail for safety and population‐level impact, with social harms monitoring and household surveys conducted to evaluate coverage and linkage, as reported elsewhere 17, 18, 19, 20. Use of distributed kits was confirmed for 275,419 (53.0%) by return of used kits, with country‐level data for this variable being 53.2% (86,925) in Malawi, 58.8% in Zambia (92,247) and 48.2% in Zimbabwe (96,247).

CBD models varied substantially country‐by‐country 21, 22, 23, with the Zimbabwe model being based on delivery from mobile teams that supported training and brief (three to four weeks) but intensive HIVST distribution by temporarily employed distributors. CBDs in Malawi and Zambia were employed for 12 months to provide services less intensively. Recruitment and training are summarized under methods. The number and age of recruited distributors are shown in Table 4: 46.1% of CBDAs were men, with most (55.5%) being in the 30 to 49‐year‐old age‐group. Costs per test distributed (US$7.23, US$14.58 and US$13.79 in Malawi, Zambia and Zimbabwe respectively) and evidence for likely economies of scale are detailed in an accompanying manuscript by Mangenah *et al*. in this issue 24.

**Table 4:** Demographic data (age, sex) of community‐based distributors by country

	Malawi		Zambia		Zimbabwe^a^		Total	
All	189	100%	165	100%	1599	100%	1953	100%
Men	101	53.4%	90	47.6%	709	44.3%	900	46.1%
Age group								
18 to 24	12	11.9%	8	8.9%	180	25.4%	200	22.2%
25 to 29	18	17.8%	19	21.1%	131	18.5%	168	18.7%
30 to 49	67	66.3%	49	54.4%	381	53.8%	497	55.3%
50‐plus	4	4.0%	14	15.6%	16	2.3%	34	3.8%
Women	88	46.6%	75	45.5%	890	55.7%	1053	53.9%
Age group								
18 to 24	9	10.2%	8	10.7%	195	21.9%	212	20.1%
25 to 29	26	29.5%	15	20.0%	155	17.4%	196	18.6%
30 to 49	50	56.8%	34	45.3%	504	56.6%	588	55.8%
50‐plus	3	3.4%	18	24.0%	37	4.2%	58	5.5%

a Zimbabwe used a “campaign‐style” distribution model with temporary distributors trained and employed for six weeks for distribution in their respective local community, while community‐based distributors in Malawi and Zambia were employed for 12 months covering larger geographic areas of distribution.

### 3.4 Integrated HTS model offering clients the choice between standard HTS and HIVST

In Malawi and Zimbabwe, HIVST was introduced at PSI HTS centres and mobile outreach, with 2199 (1.3%) of kits in Malawi, and 52,254 (19.7%) of kits in Zimbabwe to July 2018 distributed using this model (Table 2). Men made up half of HIVST clients (51.9% and 48.2% in Malawi and Zimbabwe).

Clients opting for HIVST and those preferring standard HTS clients are detailed together with the yield of positive HIV/HIVST results for 12 delivery sites in Table 5, which includes a further four months of delivery at scale (to November 2018 during which time HIVST distribution doubled under this model). Of the 119,991 individuals who accessed testing at 11 outreach and one fixed site, 101,624 (84.7%) opted for HIVST, with no difference in choice by sex. Very high proportions of individual testers: 92.4% (bus‐terminus), 92.3% (HTS centre), 91.9% (workplace), 91.5% (truck stop) chose HIVST over provider‐delivered standard testing (Table 5). When HTS and HIVST was offered at household level, 61.9% opted for HIVST.

**Table 5:** HIVST integration with HTS at 11 outreach and 1 fixed sites, Zimbabwe, HIVST was offered as alternative to provider delivered testing, 11 months of implementation to November 2018

Targeting site type	HIVST					Provider delivered testing									Total tested including HIVST	Positivity rate	Positivity rate
	Males screened negative by HIVST	Females screened negative by HIVST	Males screened positive by HIVST	Females screened positive by HIVST	Total males and females opting for HIVST	Males provider tested	Females provider tested	Total (males and females) opting for Provider testing	Proportion of total tested opting for HIVST, %	Males confirmed Positive after HIVST	Females confirmed Positive after HIVST	Males provider tested Positive Identified	Females provider Tested Positive Identified	Total positive identified		Including HIVST, %	Excluding HIVST testers, %
Bus terminus	1243	633	40	47	1963	97	64	161	92.4	17	17	0	3	37	2124	1.70	23.00
Commercial farms	2089	1257	121	112	3579	578	548	1126	76.1	31	43	32	30	136	4705	2.90	12.10
Formal mine	689	173	34	15	911	168	53	221	80.5	4	2	8	0	14	1132	1.20	6.30
Household testing	4705	6608	572	1057	12,942	3678	4281	7959	61.9	218	326	535	669	1748	20,901	8.40	22.00
Informal mines	932	245	70	37	1284	325	95	420	75.4	16	6	9	11	42	1704	2.50	10.00
Informal settlements	277	314	27	27	645	31	40	71	90.1	7	10	2	3	22	716	3.10	31.00
Workplace‐market place	1935	1852	88	106	3981	165	185	350	91.9	25	34	1	6	66	4331	1.50	18.90
Resettled farms	439	503	43	52	1037	131	240	371	73.7	7	14	6	9	36	1408	2.60	9.70
Rural shopping centres	5406	4237	298	286	10,227	977	888	1865	84.6	99	107	39	46	291	12,092	2.40	15.60
HTS centres	22,289	27,887	474	880	51,530	1821	2485	4306	92.3	241	486	194	261	1182	55,836	2.10	27.50
Truck stops	212	122	11	12	357	19	14	33	91.5	0	1	2	1	4	390	1.00	12.10
Urban shopping centres	6974	5476	317	401	13,168	818	666	1484	89.9	80	117	31	33	261	14,652	1.80	17.60
Totals	47,190	49,307	2095	3032	101,624	8808	9559	18,367	84.7	745	1163	859	1072	3839	119,991	3.20	20.90

HIVST, HIV self‐testing.

Among those self‐testing, 1908 (859 men and 1072 women) were newly diagnosed with HIV. Provider HTS clients had a substantially higher HIV prevalence (10.2% positive) than self‐testers (1.9% positive).

### 3.5 Other models of distribution

Other models (Table 2) included public sector facilities in Zambia (45.8% men) and Zimbabwe (29.0% men) and workplace distribution (9850 kits) in Malawi and Zimbabwe, with over 66.4% and 58.9% of HIVST kits taken by men.

A total of 23,561 tests were distributed to men reached with mobilization for VMMC in Malawi (1327), Zambia (15,092) and Zimbabwe (7142). Referral tracking data from Zimbabwe showed that 40.2% of males who had received HIVST kits prior to VMMC went on to be circumcised.

## 4 Discussion

STAR is the largest evaluation of HIVST implementation to date. With 628,705 kits distributed in Malawi, Zambia and Zimbabwe within 15 months of introducing HIVST as a novel approach at community and facility‐level, acceptability was high. We used five main distribution models, although community‐based distribution accounted for 82.7% of kits distributed. Approximately half of all HIVST participants were men, with good male representation in all distribution models and age groups. A substantial minority of participants had never tested for HIV before, with this proportion higher for men (22.3%) than women (17.1%), and higher for young people (16 to 24 years: 26.9% first‐time testers) than older age‐groups. HIVST is a promising approach for reaching underserved sub‐populations who have never tested before and contributing to the realization of the UNAIDS fast‐track strategy.

Consistent with previous reports 15, 16, 22, 23, all distribution models had high male participation in each country. Strategies that provide men with greater coverage of HIV testing and care are urgently needed both to address the disproportionately high testing gap and mortality from HIV in men, and also to reduce risk of onward transmission of HIV 3, 4, 5, 6, 7, 25. Peak HIV prevalence for men in southern Africa is now in the 40‐ to 49‐year‐old age‐group 3, 4, 5, 6, 7, with older men among least likely to have accessed standard HIV testing services 5, 6, 7. Older men appear relatively receptive to HIVST, however, as evidence by the data reported here as well as from implementation studies from Kenya, Lesotho and Zimbabwe 15, 26, 27, 28. For adolescent boys, HIVST can provide the first opportunity to test without fear of judgement from parents and healthcare workers 14, explaining the high uptake among this age group when HIVST was offered at community level. Thirty five percent of adolescent boys accepting self‐testing were first‐time testers in the STAR project in Malawi 14.

The STAR CBD distribution model was evaluated using household surveys, with uptake providing a measure of acceptability. Community‐level coverage of HIVST was 42.5% of all surveyed adults in rural Malawi 29, and 50.3% in rural Zimbabwe 19. This type of community‐based HIVST distribution could then contribute to activities such as national HIV testing campaigns, targeted “catch‐up” campaigns in districts with low testing coverage, and as a way of providing ongoing or periodical HIV testing access in remote communities. Costs (range US$7.23 per kit distributed in Malawi, to US$14.58 in Zambia) and affordability of the CBD model are discussed in the accompanying manuscript by Mangenah *et al*. 24, and the potential to devolve HIVST further through community‐led approaches is currently under investigation within STAR. Community‐led models can deliver better outcomes at or below the cost of less integrated approaches and are widely used in Africa for mass drug administration and distribution of insecticide‐treated bed nets 30.

Integrating HIVST into routine HTS services and in clinical settings, where access barriers may preclude testing by everyone that requires it, also shows promise with over 80% of men and women accepting HIVST when offered as an alternative to provider‐delivered HIV testing. Our data, alongside that from alternative models of integrated facility‐based HIVST 31, suggest that HIVST can contribute substantially to comprehensive provider‐initiated HTS in high volume and congested public sector clinics 31. This model could be expanded to public sector healthcare facilities more widely, especially where the current testing capacity is limited or poorly implemented 31. We also show marked preference for HIVST in all fixed and outreach HTS sites where HIVST was offered as alternative to standard HTS. Preference for HIVST was most pronounced when queuing was needed to access standard HTS, but was also apparent in home‐based testing services where 61.9% of clients who tested opted for HIVST, supporting other suggestions that HIVST is generally preferred by many Africans 14, 16.

Integrated HIVST offers potential efficiency gains, minimizing time commitments for clients and streamlining management for providers in part due to much lower HIV prevalence in those opting for HIVST over HTS. This suggest a pronounced “self‐selection” as noted for sex workers – who mostly opted for standard HTS when entering dedicated sexual health services 32 before considering HIVST for subsequent tests, citing potential higher sensitivity of blood‐based provider‐delivered tests as being important to them given their high exposure. Although speculative, and needing further research to confirm this, if our data do indeed reflect self‐selection with individuals at low risk for HIV more likely to opt for HIVST, then this has a number of advantages. From the perspective of service providers, this allows for task‐sharing with low‐risk clients, allowing counsellors to focus their time on the remaining clients with a high risk of being HIV positive, and to dedicate more time on more time‐consuming testing options such as index testing and assisted partner notification.

Introducing the option of HIVST greatly increased the numbers of clients who could be served each day at rural and urban outreach services and consequently increased the number of positive cases identified per counsellor and per site at any given time 33. A further low‐cost facility‐based model (“secondary distribution”), where HIVST kits can be delivered to partners by antenatal clinic attendees and newly diagnosed PLHIV 22, 27 is being scaled‐up under STAR in Malawi and Zimbabwe and is discussed further in the accompanying manuscript relating to social harms in this issue 17.

This study has a number of limitations. This analysis is based on programmatic data from three different countries and is based on self‐reported client data, with some missing data. Data on HIVST with regards to first‐time testers, motivators and barriers to HIVST may have been prone to social desirability bias. As reporting on first‐time testing was based on a subset of self‐test users who had returned their used tests together with the questionnaire, responses might not be representative for the entire HIVST population. For the difference in HIV prevalence in our integrated HTS model, we cannot exclude alternative explanations, including that some HTS clients were obtaining confirmation of an earlier positive test or self‐test, as many clients coming in for HTS are reluctant to detail previous positive results for a variety of reasons. Finally, the results may not be generalizable to other programme contexts with less intensity of distribution or different starting attitudes and perceptions by potential HIVST users and HTS providers.

## 5 Conclusions

Men and young people in sub‐Saharan Africa contribute disproportionately to the number of PLHIV who are not aware of their status. Results from two years of large‐scale implementation of HIVST through several distribution models demonstrate how targeted roll‐out could increase coverage of HIV testing, contribute to case finding among difficult to reach priority populations, particularly among high‐risk men and young people and increase efficiency and capacity of HTS in high volume and overcrowded clinics. HIVST offers clear advantages when provided in addition to existing services, and if scaled‐up, can contribute to closing the gap towards the “first 90.”

## Competing interests

There are no competing interest.

## Authors’ contributions

K.H., E.L.C., C.J., P.S., S.G., M.M., R.C., V.M., G.S. M.N., N.M. and C.N. designed the project and its implementation. K.H., P.S. and H.A. designed the market research evaluation and M.A.M., P.M., T.K. and K.H. analysed the data with E.L.C. and C.J. providing technical guidance. K.H. completed the first draft with contributions by all co‐authors. E.L.C and C.J. provided critical review of the article.
